# Validation of a Clinical Decision Rule to Predict Abuse in Young Children Based on Bruising Characteristics

**DOI:** 10.1001/jamanetworkopen.2021.5832

**Published:** 2021-04-14

**Authors:** Mary Clyde Pierce, Kim Kaczor, Douglas J. Lorenz, Gina Bertocci, Amanda K. Fingarson, Kathi Makoroff, Rachel P. Berger, Berkeley Bennett, Julia Magana, Shannon Staley, Veena Ramaiah, Kristine Fortin, Melissa Currie, Bruce E. Herman, Sandra Herr, Kent P. Hymel, Carole Jenny, Karen Sheehan, Noel Zuckerbraun, Sheila Hickey, Gabriel Meyers, John M. Leventhal

**Affiliations:** 1 Division of Emergency Medicine, Ann & Robert H. Lurie Children’s Hospital of Chicago, Chicago, Illinois; 2 Department of Pediatrics, Northwestern University Feinberg School of Medicine, Chicago, Illinois; 3 Department of Bioinformatics and Biostatistics, School of Public Health and Information Sciences, University of Louisville, Louisville, Kentucky; 4 Department of Bioengineering, J.B. Speed School of Engineering, University of Louisville, Louisville, Kentucky; 5 Division of Child Abuse Pediatrics, Ann & Robert H. Lurie Children’s Hospital of Chicago, Chicago, Illinois; 6 Mayerson Center for Safe and Healthy Children, Cincinnati Children’s Hospital, Department of Pediatrics, University of Cincinnati College of Medicine, Cincinnati, Ohio; 7 Department of Pediatrics, UPMC Children’s Hospital of Pittsburgh, Pittsburgh, Pennsylvania; 8 Division of Emergency Medicine, Cincinnati Children’s Hospital Medical Center, Cincinnati, Ohio; 9 Department of Pediatrics, The Ohio State University, Nationwide Children’s Hospital, Columbus; 10 Department of Pediatrics, University of California San Diego School of Medicine, La Jolla; 11 Department of Emergency Medicine, University of California, Davis Medical Center, Sacramento; 12 Department of Pediatrics, University of Chicago, Chicago, Illinois; 13 Division of Pediatric Emergency Medicine, Advocate Children's Hospital, Oak Lawn, Illinois; 14 Division of General Pediatrics, Perelman School of Medicine at the University of Pennsylvania, Philadelphia; 15 Norton Children’s Pediatric Protection Specialists Affiliated with the University of Louisville School of Medicine, Louisville, Kentucky; 16 Department of Pediatrics, University of Utah School of Medicine, Salt Lake City; 17 Division of Pediatric Emergency Medicine, University of Louisville, Louisville, Kentucky; 18 Department of Pediatrics, Penn State College of Medicine, Penn State Health Children’s Hospital, Hershey, Pennsylvania; 19 Department of Pediatrics, University of Washington, Seattle Children’s Hospital, Seattle; 20 Department of Social Work, Ann & Robert H. Lurie Children’s Hospital of Chicago, Chicago, Illinois; 21 Department of Pediatrics, Yale School of Medicine, New Haven, Connecticut

## Abstract

**Importance:**

Bruising caused by physical abuse is the most common antecedent injury to be overlooked or misdiagnosed as nonabusive before an abuse-related fatality or near-fatality in a young child. Bruising occurs from both nonabuse and abuse, but differences identified by a clinical decision rule may allow improved and earlier recognition of the abused child.

**Objective:**

To refine and validate a previously derived bruising clinical decision rule (BCDR), the TEN-4 (bruising to torso, ear, or neck or any bruising on an infant <4.99 months of age), for identifying children at risk of having been physically abused.

**Design, Setting, and Participants:**

This prospective cross-sectional study was conducted from December 1, 2011, to March 31, 2016, at emergency departments of 5 urban children’s hospitals. Children younger than 4 years with bruising were identified through deliberate examination. Statistical analysis was completed in June 2020.

**Exposures:**

Bruising characteristics in 34 discrete body regions, patterned bruising, cumulative bruise counts, and patient’s age. The BCDR was refined and validated based on these variables using binary recursive partitioning analysis.

**Main Outcomes and Measures:**

Injury from abusive vs nonabusive trauma was determined by the consensus judgment of a multidisciplinary expert panel.

**Results:**

A total of 21 123 children were consecutively screened for bruising, and 2161 patients (mean [SD] age, 2.1 [1.1] years; 1296 [60%] male; 1785 [83%] White; 1484 [69%] non-Hispanic/Latino) were enrolled. The expert panel achieved consensus on 2123 patients (98%), classifying 410 (19%) as abuse and 1713 (79%) as nonabuse. A classification tree was fit to refine the rule and validated via bootstrap resampling. The resulting BCDR was 95.6% (95% CI, 93.0%-97.3%) sensitive and 87.1% (95% CI, 85.4%-88.6%) specific for distinguishing abuse from nonabusive trauma based on body region bruised (torso, ear, neck, frenulum, angle of jaw, cheeks [fleshy], eyelids, and subconjunctivae), bruising anywhere on an infant 4.99 months and younger, or patterned bruising (TEN-4-FACESp).

**Conclusions and Relevance:**

In this study, an affirmative finding for any of the 3 BCDR TEN-4-FACESp components in children younger than 4 years indicated a potential risk for abuse; these results warrant further evaluation. Clinical application of this tool has the potential to improve recognition of abuse in young children with bruising.

## Key Points

**Question:**

Can bruising characteristics distinguish abusive from nonabusive injury in young children?

**Findings:**

In this cross-sectional study of 2161 children younger than 4.0 years, the bruising clinical decision rule (BCDR) was 96% sensitive and 87% specific for distinguishing abusive from nonabusive trauma in young children based on the characteristics of their bruising.

**Meaning:**

According to these findings, young children with an affirmative finding for any of the 3 components of the BCDR are at increased risk of abuse and warrant further evaluation.

## Teaser

This cross-sectional study assesses a previously derived bruising clinical decision rule for identifying children at risk of having been physically abused.

## Introduction

Bruising is the most common injury from physical child abuse^1,2^ and the most common injury to be overlooked or misdiagnosed as nonabusive before an abuse-related fatality or near-fatality in a young child.^3,4,5^ Several studies^4,5,6^ identified bruises as the preceding injury to abusive head trauma. Failure to recognize bruising caused by physical child abuse is a missed opportunity and an error in medical decision-making that contributes directly to poor patient outcomes.^7,8,9,10^ Published evidence confirms that measurable differences exist between bruising from nonabusive and abusive injury in infants and young children.^10,11,12,13,14,15,16,17,18,19,20,21,22,23^ An evidence-based screening tool may prevent these high-stakes failures.

Modeling these differences into an easy-to-use clinical decision rule may prevent further abuse through improved recognition. Clinical decision rules are point-of-care tools that can improve decision-making accuracy^24,25^ and health outcomes for children. Such a rule is especially critical for infants and young children who are at the highest risk of serious, potentially fatal abuse^2^ and who are too young or afraid to state what happened.

Pierce et al^15^ previously derived a bruising clinical decision rule (BCDR) named the TEN-4 (bruising to the torso, ear, or neck or any bruising on an infant <4 months of age), which is applicable to children younger than 4 years who have bruising. The TEN-4 rule exhibited high sensitivity and specificity but was derived from a small retrospective sample collected from a single pediatric intensive care unit. Information on bruising in discrete regions of the face and patterns of bruises was not available. Therefore, the objective of this study was to address these limitations and refine and validate the BCDR with the goal of establishing the first clinically sensible evidence-based screening tool to distinguish bruises caused by physical child abuse from those caused by nonabuse.

## Methods

### Study Design, Setting, and Participants

We conducted a prospective, observational, cross-sectional study of patients younger than 4.0 years with bruising. The children presented to 1 of the following 5 pediatric emergency departments: Ann & Robert H. Lurie Children’s Hospital of Chicago, Cincinnati Children’s Hospital Medical Center, Norton Children’s Hospital, Rady Children’s Hospital, or the University of Chicago Comer Children’s Hospital. All are tertiary care hospitals with differing patient demographics. This study was approved by the internal review boards at each site. We excluded children with injuries from motor vehicle crashes, known coagulation abnormalities, preexisting severe neuromuscular impairment resulting in spasticity, or severe extensive skin disorders because these conditions would impact expected bruise occurrence and characteristics. All other children had a deliberate skin examination performed by their emergency department practitioners to assess for bruising. Children with at least 1 bruise were eligible for enrollment. Research team members collected data on consecutive patients who underwent a consultation for possible child abuse. A waiver of authorization was granted to allow the research team to abstract data without interfering with the evaluation process. For patients not undergoing an abuse evaluation, the research team obtained written informed consent to collect data designed to parallel the extensive and detailed data collected for abuse consultations. For these patients, we used a structured sampling approach with research shifts as a feasible proxy to consecutive enrollment. Data were deidentified. Methods for our structured sampling enrollment are detailed in a previous publication.^26^ This study followed the Strengthening the Reporting of Observational Studies in Epidemiology (STROBE) reporting guideline.

Enrollment and case classification occurred from December 1, 2011, through March 31, 2016, and statistical analysis was completed in June 2020. At the outset, research staffs were trained to conduct a comprehensive history of presentation and a comprehensive, deliberate skin examination. Each skin finding was documented by photographs and recorded on a spatially mapped electronic body diagram with predefined anatomical regions. Extensive details were collected regarding each skin injury, including (1) type of skin injury (bruise or petechiae), (2) location of the skin injury on the body (34 predefined regions), and (3) whether the skin injury was patterned (bite, loop, hand slap, squeeze, grab, and multilinear).^27,28,29,30,31,32^ Total bruise count was documented. The patient’s skin tone was assessed to determine how tone affects the visibility of bruises. A set of photographs depicting 5 skin tone categories served as a reference standard. Patient skin tone was assessed by the research staff during the skin examination and categorized as fair, light, midtone, brown, or dark. The categorization was verified by the principal investigator (M.C.P.) using study photographs.

Each patient’s case information was classified as abuse, nonabuse, or indeterminate by an expert panel composed of pediatric emergency medicine and child abuse pediatrics physicians and a biomechanical engineer, all with expertise in pediatric injury. Each panelist received deidentified information in a standardized electronic format regarding history of presentation, examination findings, and imaging and laboratory results, when applicable. Each expert panelist independently reviewed the information and classified the cases based on injury and history compatibility. The interrater reliability of the panelists was high. The Kendall coefficient for the likelihood of abuse was 0.89 (95% CI, 0.87-0.91).^33^ For model refinement and validation, we used cases categorized as abuse and nonabuse and excluded indeterminate cases.

### Statistical Analysis

We calculated medians and interquartile ranges for the number of bruises and body regions bruised and compared between groups with the Wilcoxon rank sum test. We estimated differences in proportions between groups with score method 95% CIs. We also calculated the median numbers and interquartile ranges of bruises and body regions bruised and compared between groups with the Wilcoxon rank sum test. We calculated diagnostic accuracy statistics (sensitivity, specificity, positive predictive value [PPV], and negative predictive value [NPV]) at our observed prevalence of abuse and positive and negative likelihood ratios with 95% CIs for our previously derived TEN-4 BCDR. We refined the TEN-4 rule by fitting classification trees via binary recursive partitioning.^34^ We fit these models by including the following factors: the TEN (torso, ear, neck) body regions, bruising in regions not included by aggregate region of TEN, patient age in months, and whether there was patterned bruising. To optimize the sensitivity of the refined rule, we defined the cost of a false-negative prediction as 20:1 relative to a false-positive prediction.^34^ To maintain reasonable rule simplicity, we set the complexity parameter for the tree fitting to 0.05, precluding any recursive partitions of the data that failed to improve the fit of the tree by this amount or more. We calculated diagnostic accuracy statistics for the new model with 95% CIs. We internally validated the model by calculating bootstrap estimates (>10 000 loops) of the diagnostic accuracy statistics with nonparametric 95% CIs. All analyses were conducted in the open-source R software environment and the R library rpart (R Foundation for Statistical Computing).^35,36^

## Results

### Study Recruitment and Patient Characteristics

**Figure 1.**
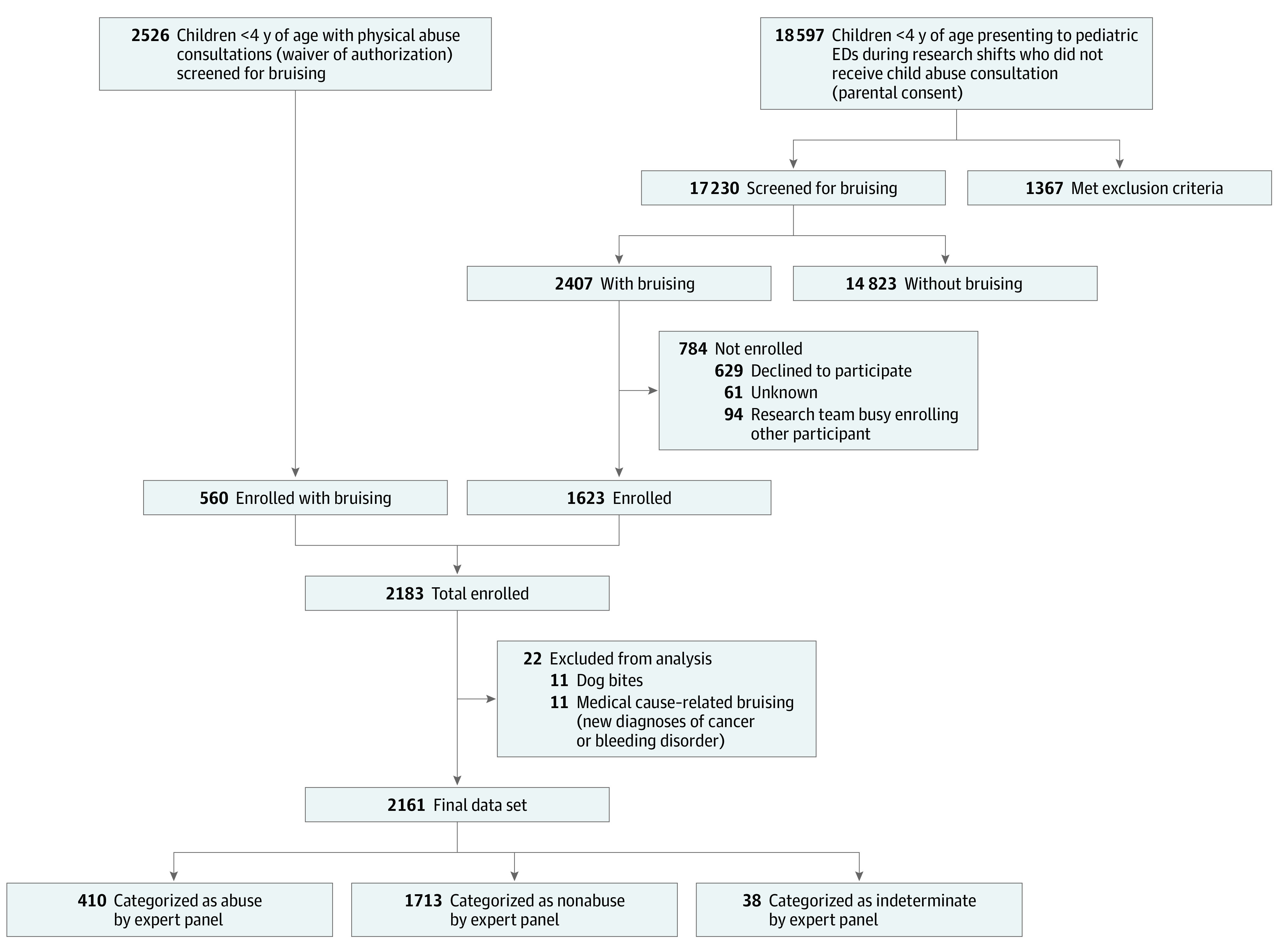
Flow of Patient Enrollment Screening for bruising and patient eligibility with final enrollment counts and expert panel categorization of abuse and nonabuse. ED indicates emergency department.

A total of 21 123 children were screened for bruising, and 2161 patients (mean [SD] age, 2.1 [1.1] years; 1296 [60%] male; 1785 [83%] White; 1484 [69%] non-Hispanic/Latino) were enrolled (Table). Abused patients were younger (mean [SD] age, 1.6 [1.2] years) and more likely to be of a race other than White (109 [27%] vs 260 [15%]), be of non-Hispanic/Latino ethnicity (349 [85%] vs 1108 [65%]), and have government (298 [73%] vs 783 [46%]) or no (26 [6%] vs 38 [2%]) medical insurance than nonabuse patients. Study flow, exclusions, and categorization outcomes are shown in Figure 1.

**Table.** Distribution of Clinical and Demographic Characteristics of Study Sample by Injury Classification^a^

Characteristic	Overall (N = 2161)	Abuse (n = 410)	Nonabuse (n = 1713)	Indeterminate (n = 38)
Age group, y				
0-0.99	349 (16)	160 (39)	180 (11)	9 (24)
1-1.99	610 (28)	88 (21)	509 (30)	13 (34)
2-2.99	644 (30)	97 (24)	537 (31)	10 (26)
3-3.99	558 (26)	65 (16)	487 (28)	6 (16)
Male sex	1296 (60)	243 (59)	1029 (60)	24 (63)
White race	1785 (83)	301 (73)	1453 (85)	31 (82)
Non-Hispanic/Latino ethnicity	1484 (69)	349 (85)	1108 (65)	27 (71)
Insurance				
Government	1102 (51)	298 (73)	783 (46)	21 (55)
Private	971 (45)	77 (19)	883 (52)	11 (29)
None	68 (3)	26 (6)	38 (2)	4 (11)
Unknown or not reported	20 (1)	9 (2)	9 (1)	2 (5)
Reason for care				
Medical	990 (46)	123 (30)	855 (50)	12 (32)
Trauma	952 (44)	93 (23)	847 (49)	12 (32)
Child abuse evaluation	219 (10)	194 (47)	11 (1)	14 (37)
GCS score <15	58 (3)	46 (11)	11 (1)	1 (3)
ED discharge disposition				
Home from ED	1685 (78)	168 (41)	1491 (87)	26 (68)
Admit to hospital	283 (13)	85 (21)	191 (11)	7 (18)
Foster care	101 (5)	96 (23)	3 (0)	2 (5)
Admit to PICU	71 (3)	54 (13)	14 (1)	3 (8)
Fatalities	12 (1)	12 (3)	0	0
Skin tone				
Fair	500 (23)	126 (31)	364 (21)	10 (26)
Light	201 (9)	27 (7)	168 (10)	6 (16)
Mid	1296 (60)	197 (48)	1080 (63)	19 (50)
Brown	108 (5)	35 (9)	71 (4)	2 (5)
Dark	56 (3)	25 (6)	30 (2)	1 (3)
Bruise count, median (IQR)				
All ages	3 (1-6)	7 (3-11)	3 (1-5)	2.5 (2-5)
Age 0-0.99 y	2 (1-5)	5 (3-10)	1 (1-2)	2 (1-2)
Age 1-1.99 y	2 (1-4)	6 (3-11)	2 (1-4)	3 (3-6)
Age 2-2.99 y	3 (2-6)	8 (5-14)	3 (2-5)	2 (1-2)
Age 3-3.99 y	4 (2-7)	9 (4-14)	4 (2-6)	4.5 (2-8)

Abbreviations: ED, emergency department; GCS, Glasgow Coma Scale; IQR, interquartile range; PICU, pediatric intensive care unit.

^a^ Data are presented as number (percentage) of children unless otherwise indicated.

### Bruising Characteristics

**Figure 2.**
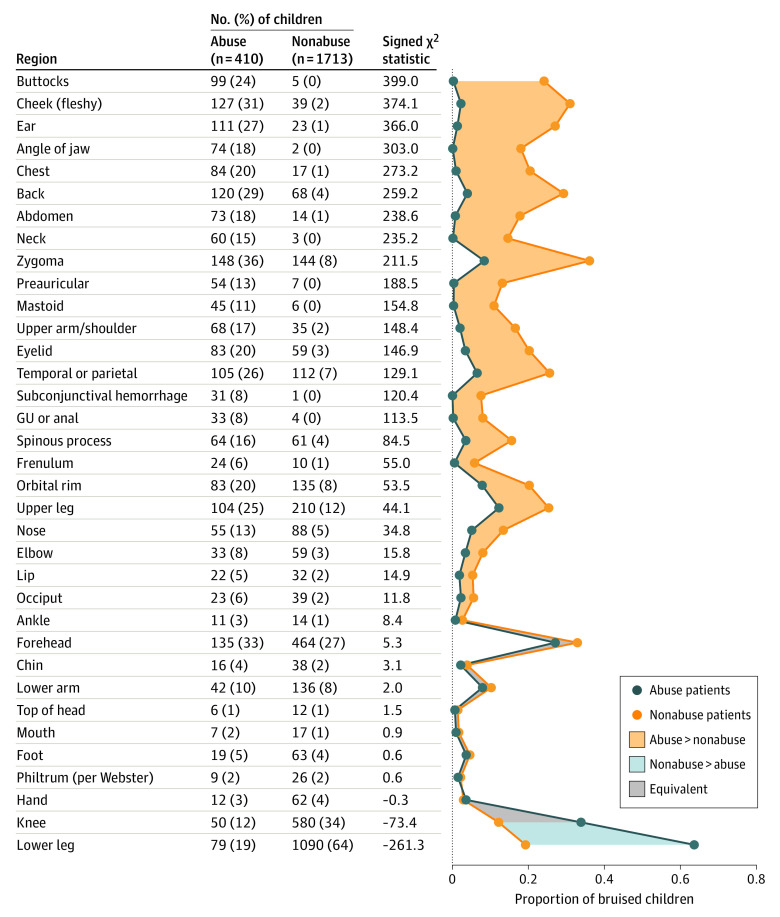
Occurrence of Bruises in Body Regions The χ^2^ statistics were derived from tests of bruising in each body region against abuse status, with signs defined by direction of association (positive is defined as abuse greater than nonabuse). Body regions are sorted in order of discriminatory power for abuse, defined by the signed χ^2^ statistic. GU indicates genitourinary.

The median number of bruises per patient was 3 (range, 1-45). Abuse patients had higher bruise counts than nonabuse patients overall and within each year of age (Table). Patients with fair-toned skin had a higher bruise count (median, 4; IQR, 2-7) than patients with the 4 darker tones (median, 3; IQR, 1-6) (eTable 1 in the Supplement). The frequency of bruising for all 34 body regions for abuse and nonabuse patients is provided in Figure 2 in descending order of ability to discriminate abuse from nonabuse. Patterned bruising was relatively uncommon (169 cases [8.0%]) but far more common in abuse (159 [38.8%]) than in nonabuse patients (10 [0.6%]). The differences in the regions affected between abuse and nonabuse patients were substantial and are visually depicted on our body diagram composites for each of the 4 age groups (eFigure 1 in the Supplement).

### TEN-4 Performance and New BCDR Derivation

The previously derived TEN-4 rule identified 331 of 410 abuse patients correctly and was also positive for 170 of 1713 nonabuse patients, producing a sensitivity of 80.7% (95% CI, 76.5%-84.4%) and a specificity of 91.1% (95% CI, 89.7%-92.4%).^15^ The PPV of the TEN-4 rule was 68.5% (95% CI, 64.1%-72.6%), and the NPV was 95.2% (95% CI, 94.0%-96.1%). Thus, the sensitivity of the TEN-4 rule was unacceptably low, missing 19% of abuse patients, which indicated a need for refinement.

To refine the TEN-4 BCDR, the classification tree we fit to these new data preserved the TEN regions (torso, which includes chest, abdomen, back, buttocks, and genitourinary area; ear; and neck), modified the infant age threshold from younger than 4.0 months to 4.99 months and younger, included patterned bruising (as defined above), and added the following body regions: angle of the jaw, cheeks (fleshy), eyelids, and subconjunctivae. We empirically added the frenulum as a region to the rule. The frenulum substantially overlapped with bruising in other BCDR regions (overlap occurred in 24 of 34 instances of frenulum injury [71%]), incurred a low cost to specificity (10 additional false-positive results; 0.6% specificity loss), captured a misclassified abuse patient admitted to the pediatric intensive care unit, and accounted for 2 fatalities. In addition, frenulum bruising corresponded to the highest fatality rate associated with the occurrence of bruising in any body region (2 of 34 [6%]).

**Figure 3.**
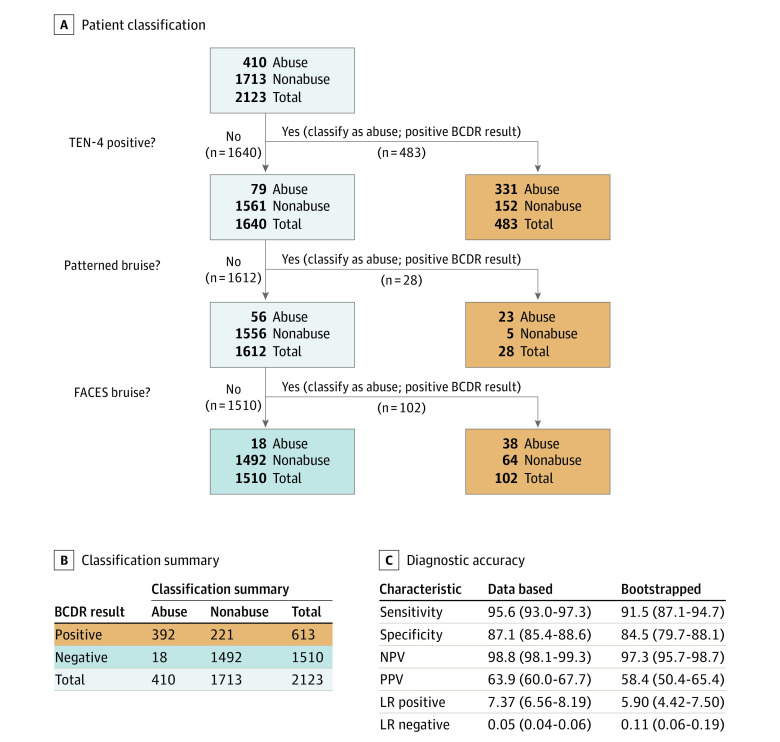
Bruising Clinical Decision Rule (BCDR) A, Classification of patients into abuse and nonabuse groups based on dichotomous independent variables (ie, TEN-4 [bruising to the torso, ear, and/or neck or any bruising on an infant <4 months of age]), positive, patterned bruise, or FACES [frenulum, angle of jaw, cheeks (fleshy), eyelids, subconjunctivae] bruise) according to an expert panel. The BCDR results were positive for 613 patients, of whom 392 were abuse patients and 221 nonabuse patients. The BCDR results were negative in 1713 patients, of whom 1492 were nonabuse patients and 18 abuse patients. B, Classification summary. C, Diagnostic accuracy statistics. Data in parentheses are 95% CIs. LR indicates likelihood ratio; NPV, negative predictive value; PPV, positive predictive value.

We named the refined BCDR as TEN-4-FACESp, for torso, ear, neck (TEN), frenulum, angle of jaw, cheeks (fleshy), eyelids, subconjunctivae (FACES), and patterned (p). The 4 represents any bruising anywhere to an infant 4.99 months or younger. The rule applies only to children *with bruising* who are younger than 4.0 years. A positive response for any of these components signals a classification of abuse. The refined BCDR had a sensitivity of 95.6% (95% CI, 93.0%-97.3%), a specificity of 87.1% (95% CI, 85.4%-88.6%), an NPV of 98.8% (95% CI, 98.1%-99.3%), and a PPV of 63.9% (95% CI, 60.3%-67.7%). Additional characteristics describing the diagnostic accuracy of the TEN-4-FACESp BCDR are shown in Figure 3. Bootstrap-derived test characteristics of sensitivity, specificity, NPV, and PPV were slightly reduced from the estimates calculated from the full data set (Figure 3). Test characteristics did not substantially vary by skin tone (eTable 1 in the Supplement). Cases misclassified by the TEN-4-FACESp are detailed in eTables 2 through 4 and eFigure 2 in the Supplement.

## Discussion

This prospective, multicenter study refined and validated the previously derived TEN-4 BCDR. The TEN-4-FACESp BCDR yielded a high sensitivity of 96% and an acceptable specificity of 87%. The intent of the validated BCDR is not to diagnose abuse but to function as a screening tool to improve the recognition of potentially abused children with bruising who require further evaluation, while minimizing overcapturing of children whose bruises are nonabuse related. As with other injury-focused research, the BCDR serves only to inform and never to supplant clinical judgment.^37,38,39,40,41,42^

The strongest differentiator of bruising characteristics between abusive and nonabusive bruises was body region bruised. The torso, ear, and neck alone correctly identified 81% of abuse patients. Body regions with bruises on more than 100 abuse patients included the fleshy part of the cheeks, the back, and the ears. The most specific regions for abuse, with 5 or fewer false-positive findings, were the buttocks, genitourinary area or anus, angle of the jaw, neck, and subconjunctival hemorrhage. Many regions indicative of abuse (such as the temporoparietal area) were not included in the body region component of the final BCDR because the patients were already captured through a different more indicative body region or inclusion would have been too costly to specificity. For example, the zygoma and forehead were common in abuse patients, but these regions also had high rates of occurrence in nonabusive injury and did not distinguish abusive from nonabusive bruising. Consistent with the study findings, forces applied to the body from physical assault of a child (eg, grabbing the torso, neck, or face)^14,27,29,32,43^ would be expected to result in bruising to regions of the body that differ from those associated with nonabusive injury (eg, falling forward onto the ground or falling off furniture). Several other studies^10,11,12,14,15,16,18,19,20,21,22,23,44,45^ also found bruising to the regions in the BCDR to be highly associated with abuse. Notably, bruising to the ears, neck, angle of the jaw, cheeks (fleshy), frenulum, and buttocks were seen most often in abuse cases and rarely in nonabuse cases. Nonabusive injury most commonly led to bruising overlying bony prominences, such as to the forehead, chin, or shins (eFigure 1 in the Supplement).^17^ Other studies^14,17^ focused on nonabusive bruising had similar findings to the current study, including bruising to bony areas of the body, such as forehead or shins. These studies, in conjunction with the current study’s findings, support bruising location as an important differentiator between abuse and nonabusive injury.

Several studies found patterns or shapes of the bruising to be important for recognizing abuse.^13,28,29,46^ When a caregiver squeezes or strikes a child, causing rupture of blood vessels, this can result in the imprint of the hand or object onto the skin.^28^ Patterns can result from any object if the strike or impact causes damage to blood vessels. Consistent with other studies, the current study found patterned bruising to be highly associated with abuse, with 94% of patterned bruises observed in patients categorized as abuse.

Injuries that occur during early infancy are concerning for abuse owing to the child’s lack of gross motor skills. At this young age, each injury requires a specific and detailed history, and each injury must be plausibly accounted for in the history. There is little margin for error because infants have the greatest risk of fatal or near-fatal injury from physical abuse.^2^ Several studies have identified infant bruising to predict abuse^1,14,18,21,44,47,48^ and the presence of concomitant internal injuries.^49^ Others have found bruising to be the most common injury (or sentinel finding) in children in whom abuse was initially overlooked or underappreciated and who subsequently presented with a more severe injury.^5,6,50,51^ The current study found that bruising anywhere on an infant 4.99 months of age or younger was an important predictor of abuse, capturing patients at high risk for abuse that would have been missed if only region-specific or pattern bruising components were used. Nonabusive bruising was also observed in infants, but almost always resulted in a single bruise (Table). Others have reported a similar finding among infants.^52^ Obtaining a clear history of injury causation is imperative for informing clinical judgment to assess the plausibility of the injury.

Enrolled children presented with medical, trauma, and abuse-specific concerns representing the full spectrum of illness and severity of trauma ranging from discharge to home, hospital admission, pediatric intensive care unit admission, and fatality (Table). Many children were referred to the pediatric emergency department from primary care offices or transferred from general emergency departments, further supporting the generalizability of the study’s results across different clinical settings. Including patients without trauma complaints was important because more than two-thirds of abuse patients presented without any initial history of trauma. Other researchers have found that the caregivers of abused children often presented for care reporting a vague medical concern such as fussiness or vomiting.^5,6,53^ These findings support the importance of an informed head-to-toe skin examination in young children regardless of chief concern and a differential diagnosis guided by those findings.

Importantly, the TEN-4-FACESp BCDR is limited to children who have bruising present at the time of examination. Thus, the absence of bruising in a child precludes the use of the BCDR for identifying abuse because it is based on differences in bruising resulting from abuse vs nonabusive injury. Stated differently, the BCDR is not negative for children without bruising—it is simply not relevant for children without bruising. Alternative methods of identifying abuse not based on bruising would be needed in such circumstances.

### Limitations

This study has limitations. The study design required deliberate head-to-toe skin examinations on every child, but because of limited resources, a second confirmatory examination on all 21 123 patients screened was not feasible. Some bruises may have been missed, but the screening process was standardized. The finding of overall prevalence of bruising (14%) is higher than our previously published results of 3.5% and results from other studies in infants.^21,22,26^ Enrollment in pediatric emergency departments vs primary care clinics and an enriched sampling of potential abuse patients may account for this difference.

Skin tone is another potential limitation. We found that among children with nonabusive injury, those with the four darker skin tones had significantly lower total bruise counts than children with the lightest skin tone. This suggests that bruise visibility may have been impacted by skin tone because nonabusive injuries tend to produce bruising in predictable numbers.^10,17,21^ Sugar et al also suggested that skin tone may have played a role in bruise visibility.^21^ Consideration of skin tone is important when determining the presence or absence of bruising. Although bruises may be more difficult to see in darker-toned skin, the accuracy of the BCDR in the current study did not substantially vary across skin tones.

One other potential limitation involves the categorization of patients as abuse or nonabuse and the possibility of misclassification. The study used a 9-member panel composed of experts in child injury with extensive clinical or research expertise, because there is no true criterion standard for abuse categorization. Panelist interpretation of all injury findings and conclusions of abuse or nonabuse were made in the context of that specific child and the associated histories. Agreement among panelists was strong, and the panel was accurate in their classification of cases where the outcome was definitively known.^33^

## Conclusions

To our knowledge, this is the first validated clinical decision rule for improved recognition of physical abuse in young children with bruising. The refined and validated rule, TEN-4-FACESp, performed with high sensitivity, which is crucial for a screening tool, and yielded good specificity, which is crucial when a screening test can cause undue stress, such as might occur in abuse screening. An important next step will be implementing these findings in pediatric and general emergency departments and pediatric clinics.

## Notes

Supplement. **eTable 1.** Bruise and BCDR Characteristics by Skin Tone **eTable 2.** Characteristics of Cases Misclassified by the BCDR - 18 Abuse Patients With Negative BCDR Results (False Negatives) **eTable 3.** Bruising Characteristics of Abuse Patients Who Were Screened Negative by the BCDR (False Negatives) and the Potential Impact on BCDR if Their Missed Body Regions Were Included in the Rule **eTable 4.** BCDR Characteristics of Non-Abuse Patients Who Were Screened Positive by the BCDR (False Positives) Compared to True Positive Abuse Patients **eFigure 1.** Composite Results of Location of Bruises Due to Abuse and Non-Abuse Stratified by Body Plane and Age: A (Anterior), B (Posterior); C (Left Lateral); D (Right Lateral); E (Mandible) **eFigure 2.** Body Diagram Composite Results of Abuse Patients Who Were Screened Negative by the BCDR (False Negatives) and Non-Abuse Patients Who Were Screened Positive (False Positives) by the BCDR
