# Error in Figure Legend

**DOI:** 10.1001/jamanetworkopen.2021.30136

**Published:** 2021-09-10

In the Original Investigation titled “Validation of a Clinical Decision Rule to Predict Abuse in Young Children Based on Bruising Characteristics”^1^ published April 14, 2021, the labels indicating nonabuse patients and abuse patients were inadvertently transposed in the key for Figure 2. This article has been corrected.^1^
