# Supplementary material for: Validation of a Clinical Decision Rule to Predict Abuse in Young Children Based on Bruising Characteristics
Source: JAMA Netw Open. 2021 Apr 14;4(4):e215832. doi: 10.1001/jamanetworkopen.2021.5832 (PMC8047759; doi:10.1001/jamanetworkopen.2021.5832)
Supplement: Supplement. — eTable 1. Bruise and BCDR Characteristics by Skin Tone eTable 2. Characteristics of Cases Misclassified by the BCDR - 18 Abuse Patients With Negative BCDR Results (False Negatives) eTable 3. Bruising Characteristics of Abuse Patients Who Were Screened Negative by the BCDR (False Negatives) and the Potential Impact on BCDR if Their Missed Body Regions Were Included in the Rule eTable 4. BCDR Characteristics of Non-Abuse Patients Who Were Screened Positive by the BCDR (False Positives) Compared to True Positive Abuse Patients eFigure 1. Composite Results of Location of Bruises Due to Abuse and Non-Abuse Stratified by Body Plane and Age: A (Anterior), B (Posterior); C (Left Lateral); D (Right Lateral); E (Mandible) eFigure 2. Body Diagram Composite Results of Abuse Patients Who Were Screened Negative by the BCDR (False Negatives) and Non-Abuse Patients Who Were Screened Positive (False Positives) by the BCDR [file jamanetwopen-e215832-s001.pdf]

Supplementary Online Content

Pierce MC, Kaczor K, Lorenz DJ, et al. Validation of a clinical decision rule to predict abuse in young children based on bruising characteristics. *JAMA Netw Open*. 2021;4(4):e215832. doi:10.1001/jamanetworkopen.2021.5832

eTable 1. Bruise and BCDR Characteristics by Skin Tone

eTable 2. Characteristics of Cases Misclassified by the BCDR - 18 Abuse Patients With Negative BCDR Results (False Negatives)

eTable 3. Bruising Characteristics of Abuse Patients Who Were Screened Negative by the BCDR (False Negatives) and the Potential Impact on BCDR if Their Missed Body Regions Were Included in the Rule

eTable 4. BCDR Characteristics of Non-Abuse Patients Who Were Screened Positive by the BCDR (False Positives) Compared to True Positive Abuse Patients

eFigure 1. Composite Results of Location of Bruises Due to Abuse and Non-Abuse Stratified by Body Plane and Age: A (Anterior), B (Posterior); C (Left Lateral); D (Right Lateral); E (Mandible)

eFigure 2. Body Diagram Composite Results of Abuse Patients Who Were Screened Negative by the BCDR (False Negatives) and Non-Abuse Patients Who Were Screened Positive (False Positives) by the BCDR

This supplementary material has been provided by the authors to give readers additional information about their work.

© 2021 Pierce MC et al. *JAMA Network Open.*

eTable 1. Bruise and BCDR characteristics by skin tone. Values are median [IQR] or estimated test characteristic (95% CI).

Skin Tone	N	Abuse	Non-Abuse	Bruise Count	Sensitivity	Specificity	NPV	PPV	LR+	LR-
Fair	500	126	364	4 [2, 7]	94.4 (88.5, 97.5)	80.5 (76.0, 84.4)	97.7 (95.0, 99.0)	62.6 (55.3, 69.4)	4.84 (3.96, 5.72)	0.07 (0.04, 0.09)
Light	201	27	168	3 [1, 6]	96.3 (79.1, 99.8)	84.5 (78.0, 89.5)	99.3 (95.6, 100)	50.0 (36.9, 63.1)	6.22 (4.17, 8.27)	0.04 (0.01, 0.08)
Mid	1296	197	1080	3 [1, 5]	95.9 (91.9, 98.1)	90.0 (88.0, 91.7)	99.2 (98.3, 99.6)	63.6 (57.9, 69.1)	9.59 (8.01, 1.18)	0.05 (0.03, 0.06)
Brown	108	35	71	3 [1, 6]	94.3 (79.5, 99.0)	87.3 (76.8, 93.7)	96.9 (88.2, 99.5)	78.6 (62.8, 89.2)	7.44 (3.71, 1.17)	0.07 (0.01, 0.12)
Dark	56	25	30	3 [1, 7]	100 (83.4, 100)	76.7 (57.3, 89.4)	100 (82.2, 100)	78.1 (59.6, 90.1)	4.29 (2.23, 6.34)	0 (0, 0)
Overall	2161	410	1713	3 [1, 6]	95.6 (93.0, 97.3)	87.1 (85.4, 88.6)	98.8 (98.1, 99.3)	63.9 (60.0, 67.7)	7.37 (6.56, 8.19)	0.05 (0.04, 0.06)

© 2021 Pierce MC et al. *JAMA Network Open.*

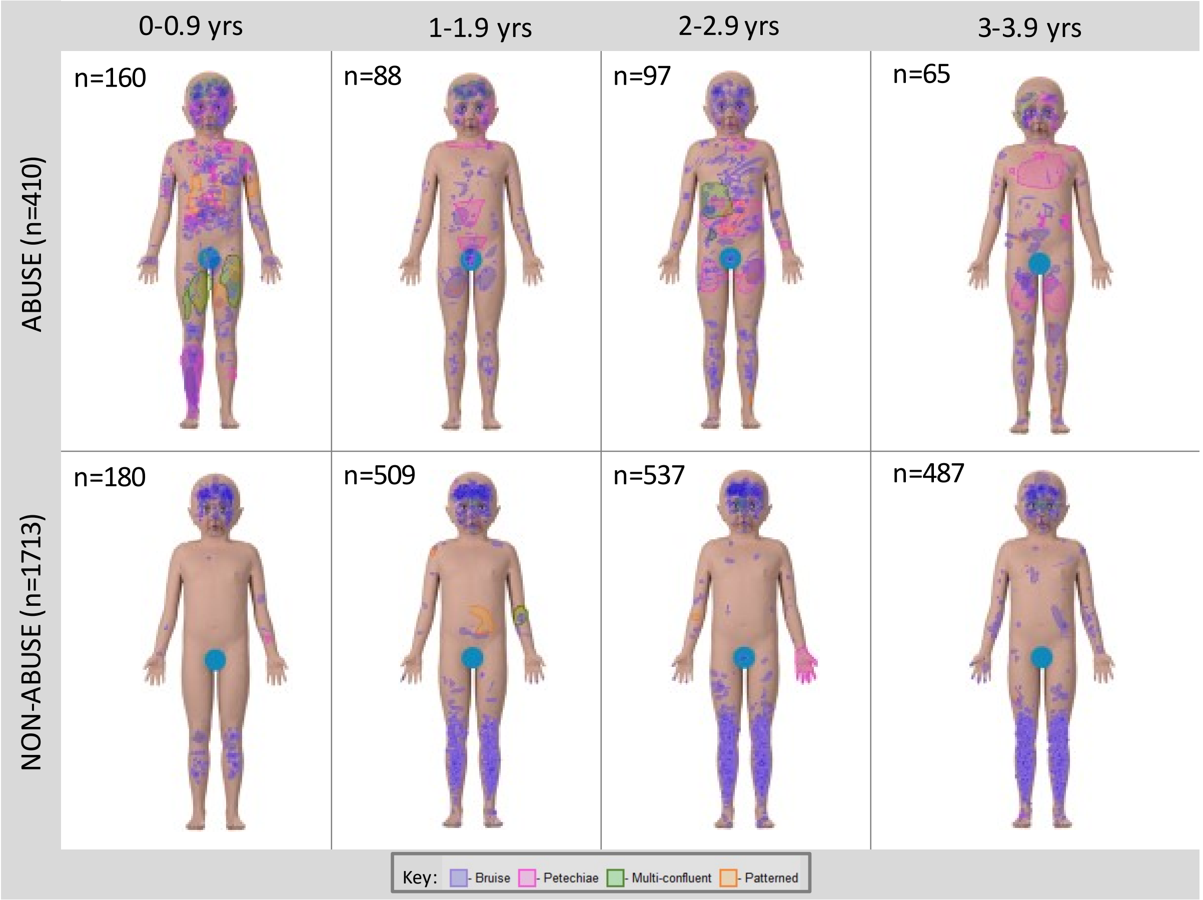

eFigure 1a. Composite results of location of bruises due to abuse and non-abuse stratified by body plane and age: Anterior

© 2021 Pierce MC et al. *JAMA Network Open.*

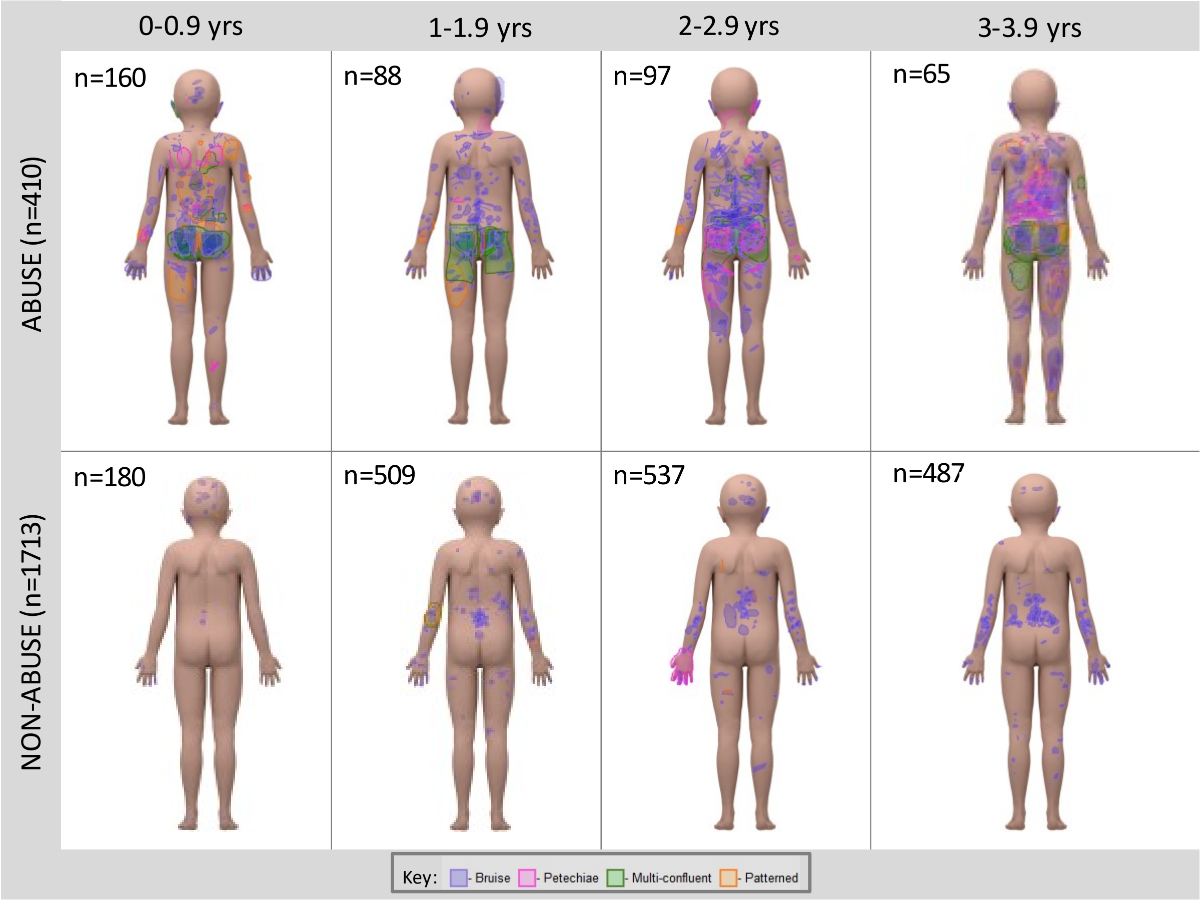

eFigure 1b. Composite results of location of bruises due to abuse and non-abuse stratified by body plane and age: Posterior

© 2021 Pierce MC et al. *JAMA Network Open.*

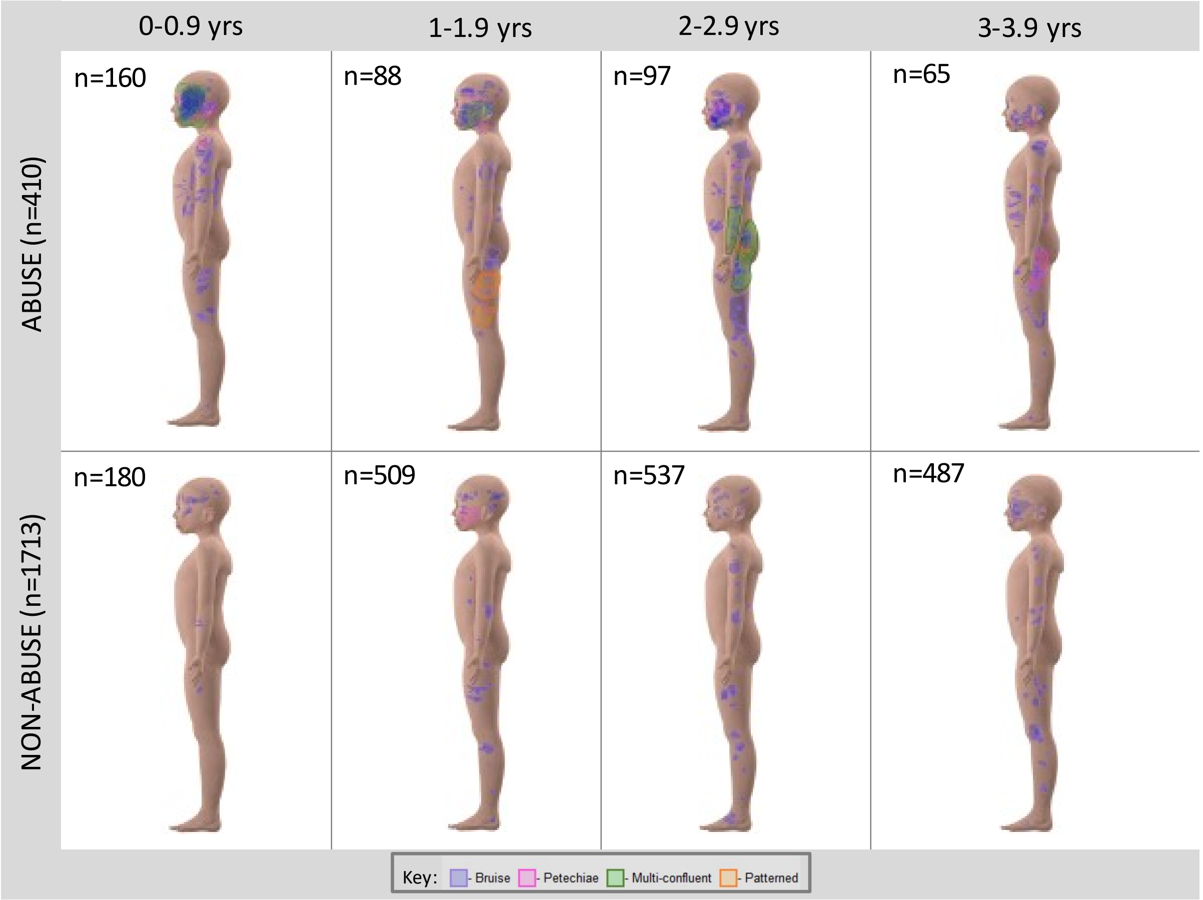

eFigure 1c. Composite results of location of bruises due to abuse and non-abuse stratified by body plane and age: Left lateral

© 2021 Pierce MC et al. *JAMA Network Open.*

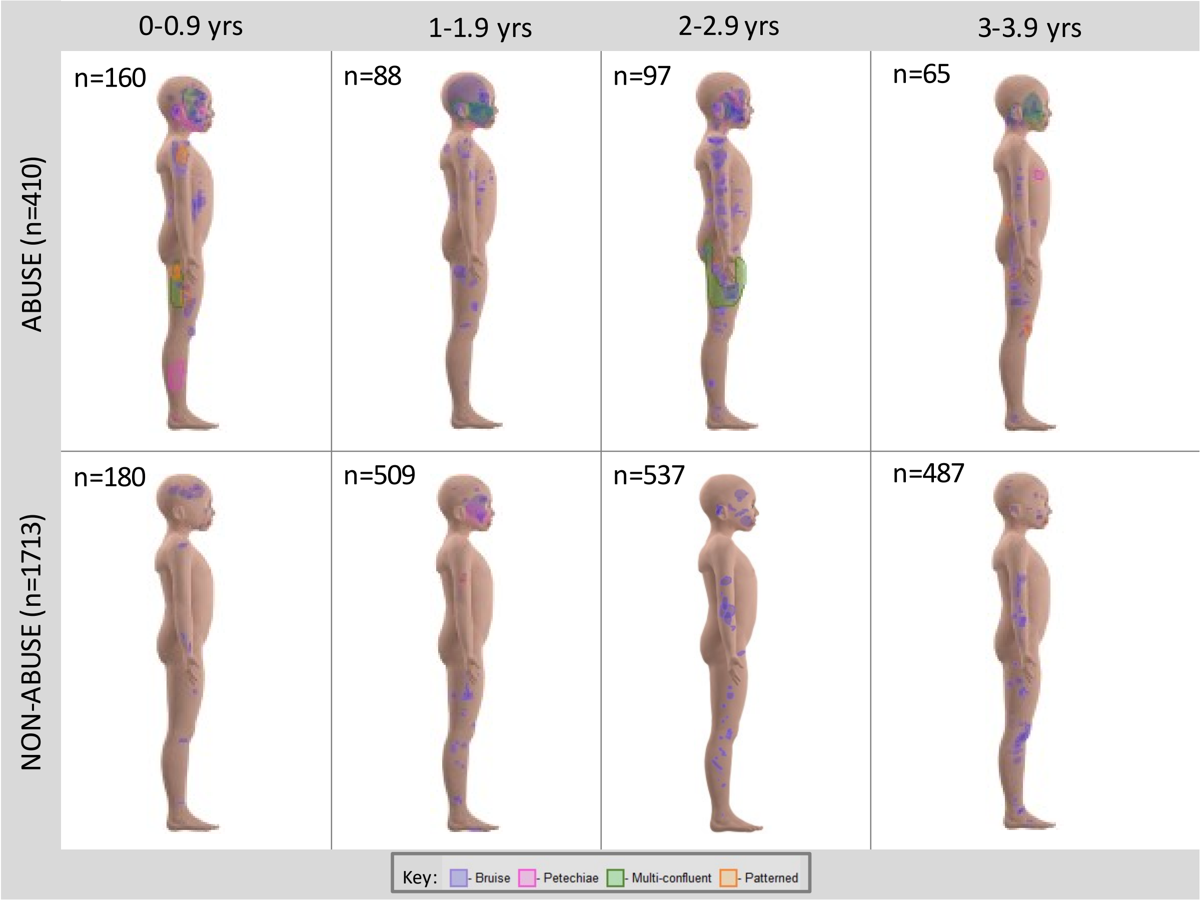

eFigure 1d. Composite results of location of bruises due to abuse and non-abuse stratified by body plane and age: Right lateral

© 2021 Pierce MC et al. *JAMA Network Open.*

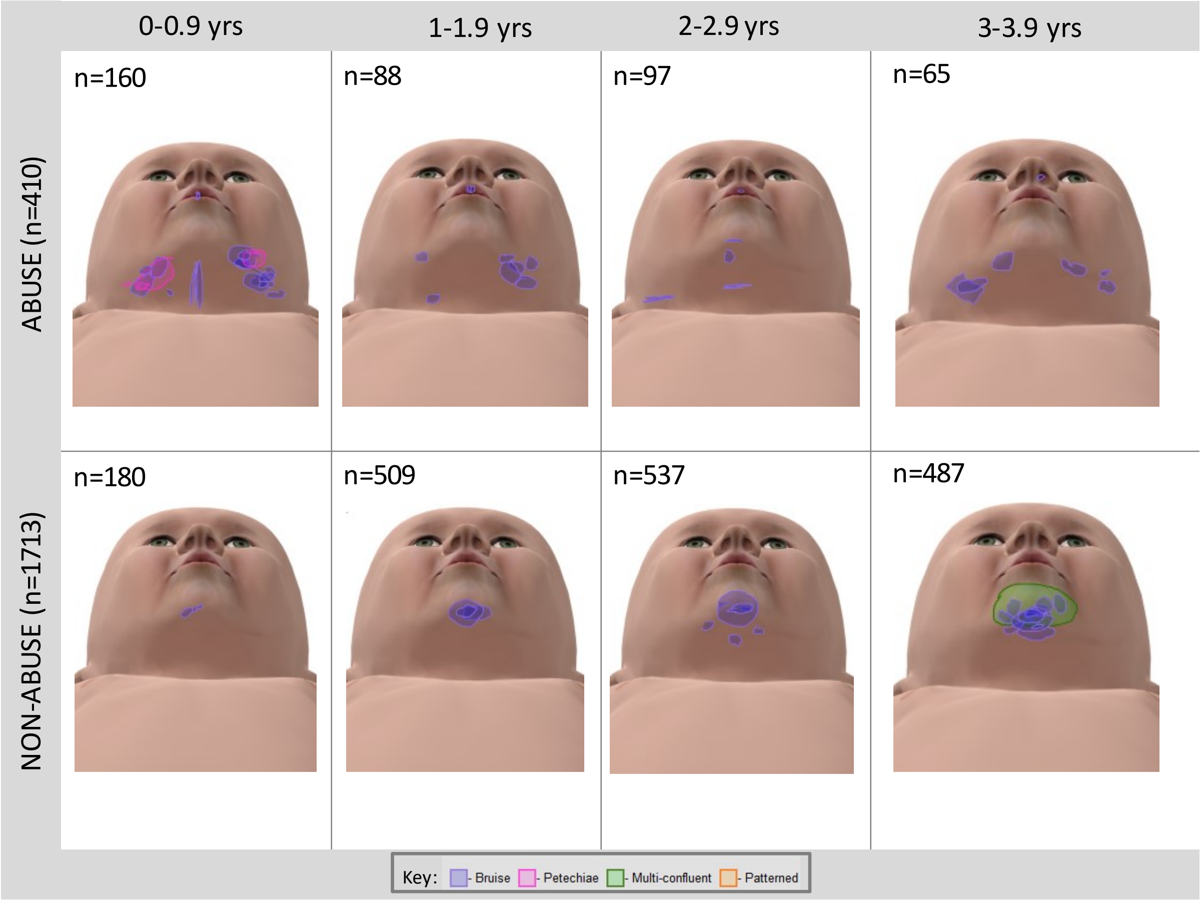

eFigure 1e. Composite results of location of bruises due to abuse and non-abuse stratified by body plane and age: Mandible

© 2021 Pierce MC et al. *JAMA Network Open.*

Misclassified Cases using the TEN-4-FACESp BCDR

The TEN-4-FACESp misclassified 18 (4%) out of the 410 abuse cases as non-abuse (eTable 2, eFigure 2). These patients exhibited bruises in 18 distinct regions of the body that were not part of the BCDR. Inclusion of any of these regions in the BCDR would come at substantial cost to specificity (eTable 3). For example, including the forehead in the rule would correctly classify an additional 9 abuse patients, but at the expense of 415 additional incorrect classifications of non-abuse patients. Out of 1710 non-abuse cases, the TEN-4-FACESp misclassified 221 (13%) as abuse. (eTable 4, eFigure 2). In 127/221 (57%) of these misclassifications, at least one of the following regions was involved - back (68 cases, 31%), eyelids (47, 21%), or cheeks-fleshy (31, 14%).

eTable 2. Characteristics of cases misclassified by the BCDR - 18 abuse patients with negative BCDR results (false negatives)

Regions of Bruises	Age	GCS	PED Disposition	Fatality
Nose; Upper leg	3 yr	15	Home	No
Foot; Nose	3 yr	15	Admit to Hospital	No
Forehead; Lower leg; Temporal/Parietal; Upper leg	3 yr	15	Home	No
Forehead; Lower leg; Nose; Orbital Rim; Temporal/Parietal	2 yr	15	Home	No
Elbow; Lower arm; Lower leg; Upper leg	2 yr	15	Home	No
Lower leg	2 yr	15	Home	No
Forehead; Lower leg; Temporal/Parietal; Upper leg	2 yr	15	Home	No
Hand	2 yr	15	Home	No
Knee	1 yr	15	Home	No
Forehead	1 yr	15	Home	No
Lower leg	1 yr	15	Home	No
Forehead	10 mo	15	Admit to PICU	No
Upper arm/Shoulder	10 mo	15	Admit to Hospital	No
Lower leg	10 mo	15	Home	No
Forehead; Knee; Occiput; Temporal/Parietal	10 mo	15	Home	No
Forehead	9 mo	5	Admit to OR	Yes
Ankle; Foot; Forehead; Top of Head; Lower leg	7 mo	15	Home	No
Forehead; Top of Head	7 mo	15	Admit to Hospital	No

© 2021 Pierce MC et al. *JAMA Network Open.*

eTable 3. Bruising characteristics of abuse patients who were screened negative by the BCDR (false negatives) and the potential impact on BCDR if their missed body regions were included in the rule

		If region added to the BCDR…			
**Body Region**	**Bruise present in a False Negative (N = 18)**	**Additional False Positives**	**Sensitivity Gain (%)**	**Specificity Loss (%)**	**Relative Cost (Spec Loss/ Sens Gain)**
Forehead	9	415	2.2	24.2	11.0
Lower leg	8	989	2.0	57.7	29.6
Temporal/parietal	4	86	1.0	5.0	5.1
Upper leg	4	188	1.0	11.0	11.2
Nose	3	71	0.7	4.1	5.7
Foot	2	58	0.5	3.4	6.9
Top of Head	2	8	0.5	0.5	1.0
Knee	2	513	0.5	29.9	61.4
Ankle	1	14	0.2	0.8	3.4
Elbow	1	50	0.2	2.9	12.0
Hand	1	56	0.2	3.3	13.4
Lower arm	1	117	0.2	6.8	28.0
Occiput	1	32	0.2	1.9	7.7
Orbital rim	1	100	0.2	5.8	23.9
Upper arm/Shoulder	1	26	0.2	1.5	6.2

© 2021 Pierce MC et al. *JAMA Network Open.*

eTable 4. BCDR characteristics of non-abuse patients who were screened positive by the BCDR (false positives) comparted to true positive abuse patients

BCDR Characteristics	False Positive (N = 221)	True Positive (N = 392)
Back*	68	120
Eyelid^	59	83
Age <= 4 mo	44	80
Cheek – fleshy^	39	127
Ear	23	111
Chest*	17	84
Abdomen*	14	73
Frenulum^	10	24
Patterned bruise	10	159
Buttocks*	5	99
GU/Anal*	4	33
Neck	3	60
Angle of jaw^	2	74
Subconjunctiva^	1	31

* Torso includes: chest, abdomen, back, buttocks and genitourinary /anal area
^FACES includes: **F**renulum, **A**ngle of jaw, **C**heek, **E**yelids, **S**ubconjunctiva

© 2021 Pierce MC et al. *JAMA Network Open.*

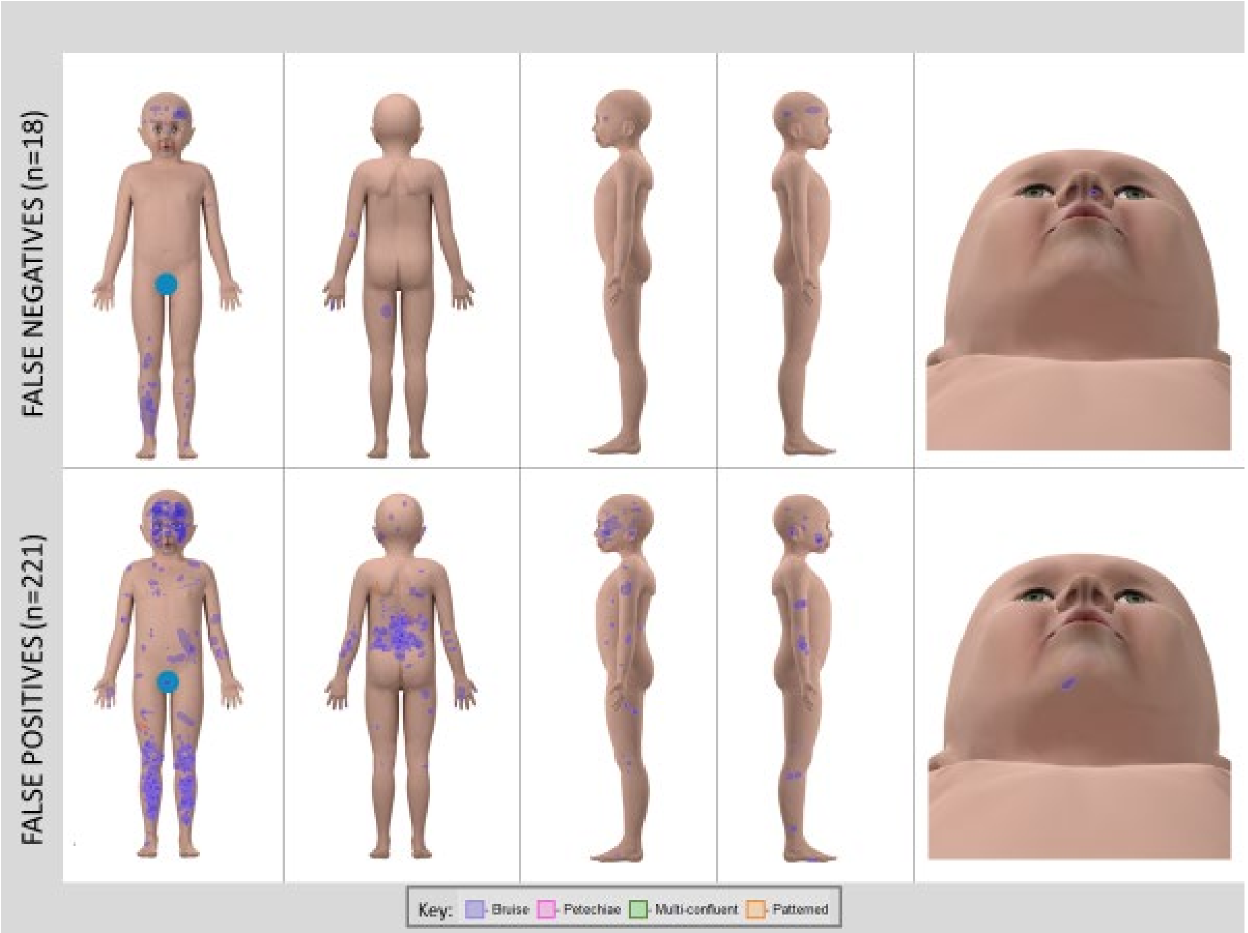

eFigure 2: Body diagram composite results of abuse patients who were screened negative by the BCDR (false negatives) and non-abuse patients who were screened positive (false positives) by the BCDR

© 2021 Pierce MC et al. *JAMA Network Open.*
